# Game and Balance Multicast Architecture Algorithms for Sensor Grid

**DOI:** 10.3390/s90907177

**Published:** 2009-09-09

**Authors:** Qingfeng Fan, Qiongli Wu, Frèdèric Magoulés, Naixue Xiong, Athanasios V. Vasilakos, Yanxiang He

**Affiliations:** 1 EcoleCentrale de Paris, Laboratory MAS, 92290, Chatenay-Malabry, France; E-Mails: qingfeng.fan@ecp.fr (Q.F.); frederic.magoule@ecp.fr (F.M.); 2 School of computer science, Wuhan University, 430079, Wuhan, China; E-Mail: yxhe@whu.edu.cn; 3 INRIA Saclay Île-de-France, 91893, Orsay Cedex, France; 4 Department of Computer Science, Georgia State University, P.O. Box 3994, Atlanta, GA 30302-3994, USA; E-Mail: nxiong@cs.gsu.edu; 5 Department of Computer and Telecommunication Engineering, University of Western Macedonia, GR 50100 Kozani, Greece; E-Mail: vasilako@ath.forthnet.gr

**Keywords:** game and balance, multicast, sensor grid

## Abstract

We propose a scheme to attain shorter multicast delay and higher efficiency in the data transfer of sensor grid. Our scheme, in one cluster, seeks the central node, calculates the space and the data weight vectors. Then we try to find a new vector composed by linear combination of the two old ones. We use the equal correlation coefficient between the new and old vectors to find the point of game and balance of the space and data factorsbuild a binary simple equation, seek linear parameters, and generate a least weight path tree. We handled the issue from a quantitative way instead of a qualitative way. Based on this idea, we considered the scheme from both the space and data factor, then we built the mathematic model, set up game and balance relationship and finally resolved the linear indexes, according to which we improved the transmission efficiency of sensor grid. Extended simulation results indicate that our scheme attains less average multicast delay and number of links used compared with other well-known existing schemes.

## 1. Introduction

A sensor grid integrates wireless sensor networks with grid infrastructures to enable real-time sensor data collection and the sharing of computational and storage resources for sensor data processing and management. It is an enabling technology for building large-scale infrastructures, integrating heterogeneous sensor, data and computational resources deployed over a wide area, to undertake complicated surveillance tasks such as environmental monitoring [1].

The sensor grid enables the collection, processing, sharing, and visualization, archival and searching of large amounts of sensor data. The vast amount of data collected by the sensors can be processed, analyzed and stored using the computational and data storage resources of the grid. The sensors can be efficiently shared by different users and applications, which can access a subset of the sensors to collect the desired type of sensor data. A sensor grid provides seamless access to a wide variety of resources in a pervasive manner [2]. Advanced techniques in artificial intelligence, data fusion, data mining, and distributed database processing can be applied to make sense of the sensor data and generate new knowledge of the environment.

In many cases the amount of data in different nodes varies considerably, the proportion between the maximum and minimum is sometimes 1:1,000,000 or even much more. These data are widely distributed in different geographical positions and dynamically updated, replicated frequently, therefore a large number of transmission is necessary [3]. The geographical scattering of sensor nodes are of interest in analyzing these data sets.

**Figure 1.**
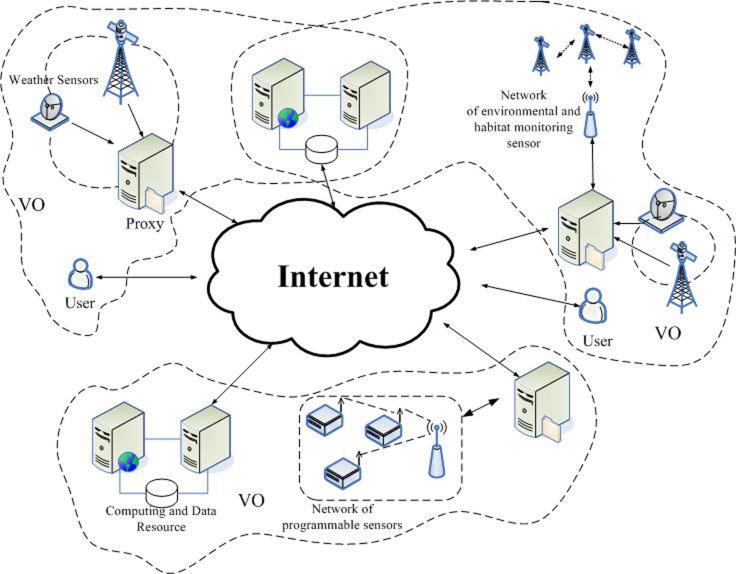
Sensor Grid Architecture.

A sensor-grid-based architecture has many applications such as environmental and habitat monitoring, healthcare monitoring of patients, weather monitoring and forecasting, military and homeland security surveillance, tracking of goods and manufacturing processes, safety monitoring of physical structures and construction sites, smart homes and offices. As shown in Figure 1, how to find a good hierarchical architecture to link the sensor grid nodes and consequently to realize an efficient data transmission is a very meaningful and challenging issue [4].

### 1.1. The Overview of Previous Algorithms

To achieve the high efficiency of the system, we proposed a set of novel Game and Balance Hierarchical Multicast Architecture Algorithms for sensor grid. The conception of multicast comes from network communication. Multicast technology is an important method of IP network data transmission. Between the senders and receivers, the system implements the link of network from one point to multi-points. According to the space relationship between one point sender and multi-points received, the system constructs optimal tree architecture for optimal data transfer. The advantage of multicast is that it can get the least using links number and shortest transfer delay, so that it promotes data transfer efficiency and decrease the possibility of network block. The most famous NICE protocol is a hierarchical multicast tree technique, which is an extendable multicast protocol that supports, from one sender to a number of receivers, low bandwidth data flow appliance.

Many well-known multicast schemes have been presented in reference listed: Double-Channel XY Multicast Wormhole Routing (DCXY) [5] uses an extension of the XY routing algorithm to set up the routing scheme. Dual-Path Multicast Routing (DPM) [6] is developed for the 2-*D* mesh. CAN-based multicast [7] is developed for the multicast applications that use the CAN [8] (Content-Addressable Network) configuration.

However, in the previous work of multicast for network communication, only one factor that affect the date transmission efficiency is considered [9], which can not be applicable for sensor grid where a lot of other factors should be considered, i.e., the data amount factor. So in this paper, we proposed an algorithm architecture for the sensor grid based on multicast concept but considering two or more factors.

### 1.2. Motivation

#### Synthetically considering the space factor and the data factor

The former hierarchical multicast schemes only consider the factor of geographical position, which means the shortest path way using the least number of links. While constructing the hierarchical multicast tree, the system often chooses the geographical central node as the cluster core or near the core. Hence it can save the transmission distance [8]. However, in sensor grid, the data quantities of different nodes are much different [10]. Usually 80% of the data often is centralized in 20% nodes; naturally these important nodes should be paid more attention to. Generally speaking, the more data the nodes have, the more data transmission will happen from the nodes [11]. If the data scale is the only factor we consider, to choose the node with larger data quantity as root or near the root would undoubtedly improve the efficiency of the data transmission.

As a result, the system should consider not only the space factor, but also the data quantity as the factor [9]. The two factors are independent with each other and related with each other. In other words, their relationship is game and balance. We try to set a group of functions in order to draw an elaborate balance between them in our to-be-presented algorithm. The basic idea goes through the whole process of constructing the hierarchical multicast tree. The space factor and data factor are two factors independent with each other, which have meaning and formation respectively; both of them tend to maximize their result. Namely the two factors game with each other. On the other hand, the two factors also co-exist in a system, common working, mutual interaction and constraint. Namely they balance with each other. We must synthetically consider the space and data factors while constructing the multicast tree.

#### The specific implementation of the algorithms

After summarizing the context of the algorithms, this subsection discusses the concrete implementation of the algorithms [12]. The motivation of this paper is to design a multicast scheme in *m*-*D* Sensor grid that can achieve not only shorter multicast delay and less resource consumption, but also the efficient data transmission.

The network is partitioned into clusters in terms of some regular Sensor grid area. After group members are initially scattered into different clusters, a tree is built to connect the cluster members within each other. The connection among different clusters is done through hooking the tree roots [13].

To construct such an architecture, a set of novel algorithms based on the *m*-*D* Sensor grid are presented:

1. Cluster formation algorithm that divides the group members into different clusters in terms of static delay distance;
2. Relative weight vectors generation algorithm that seeks the spatial central node in every cluster, calculates the space weight of every node, searches the weight of data quantity of every node, and finds the maximum;
3. The least weighted path tree algorithm that, after obtaining the space weight vector and the data quantity weight vector, builds binary simple equations, seeks linear parameters, determines the new weight vector according to the algebra sum of the two known vectors, and generates the least weighted path tree;
4. Multicast routing algorithm that efficiently dispatches the multicast packets in the group on the basis of the architecture constructed by the above three algorithms.

After checking the relative documents, this paper is one of the pioneer to use the multicast architecture for grid computing field [14], which—according to the idea from qualitative to quantitative—builds mathematic model, finds game and balance relationship, resolves linear parameter, and accurately improves the efficiency of transmission of sensor grid.

### 1.3. Organization

The architecture of this paper as follows: After describing the motivation in Section 1., the paper presents the problem in Section 1.3. and discuss how to figure out different weights in Section 2. Section 3. depicts the four sub-algorithm and the detail steps in different sub-algorithm. Section 4. describes the performance evaluation, which explains the model and result of the simulation. At last, Section 5. draws a conclusion and talks about the future work.

To improve the efficiency of data transmission in a quantitative way, the first thing we should do is to establish the mathematical model to describe the system.

The multicast group with *l* members of the system is denoted as: *G* = {*U*_0_, . . ., *U_i_*, . . ., *U*_*l*−1_}, where *i* ∈ [0, *l* − 1]. Each member can be identified by *m* coordinates: *U_i_* = (*u*_*i*,0_, . . ., *u_i,j_*, . . ., *u*_*i,m*−1_), when 0 ≤ *j* ≤ *m* − 1. For example, member *U*_0_: 2 dimension coordinates (*u*_0,0_, *u*_0,1_) as (0, 0) and member *U*_1_: 2 dimension coordinates (*u*_1,0_, *u*_1,1_) as (0, 1) etc [15].

As illustrated in Figure 4, there are two nodes *U_i_* = (*u*_*i*,0_, . . ., *u_i,j_*, . . ., *u*_*i,m*−1_) where *i* ∈ [0, *l* − 1] and *U*_*i*′_ = (*u*_*i*′,0_, . . ., *u_i′,j_*, . . ., *u*_*i′,m*−1_), where *i*′ ∈ [0, *l* − 1] and *i*′ ≠ *i*. We define *U_i_* and *U_i′_* are neighbors, if and only if *u_i,j_* = *u_i′,j_* for all *j*, except *u_i,j′_* = *u_i′,j′_* ± 1 along only one dimension *j*′. Thus, in the *m* − *D* Sensor grid, an node may have *m* to 2*m* neighbors [5].

We also define the Manhattan distance of two nodes [16]. In a 2-*D* sensor grid, the static delay distance of two nodes (*X*_0_, *Y*_0_) and (*X*_1_, *Y*_1_) is |*X*_1_ − *X*_0_| + |*Y*_1_ − *Y*_0_|. The sum of static delay distances from all the other nodes (*X_i_*, *Y_i_*) to (*X*_0_, *Y*_0_) (*i* ∈ [1, *n* − 1]) is: $f(X_{0},\;Y_{0})=\sum_{i=1}^{n-1}\left(|X_{i}-X_{0}|+|Y_{i}-Y_{0}|\right)$.

Then the question we discuss next is how to configure the space factor and the data factor. We established two weight vectors to describe the space factor and the data factor in each cluster, and the value of every item means the relative weight of every node. For example, the space weight vector of the *j* − *th* cluster is *W*′*_j_* = (*w*′_*j*,0_, . . ., *w*′_*j,i*_, . . ., *w*′_*j,n*−1_), *i* ∈ [0, *n* − 1], *n* means that there are *n* nodes in the cluster, *w*′*_j,i_* means the space weight of the node *i* within the *j* − *th* cluster; the data weight vector of the *j* − *th* cluster *W*″*_j_* = (*w*″_*j*,0_, . . ., *w*″*_j,i_*, . . ., *w*″_*j,n*−1_), *i* ∈ [0, *n* − 1], *n* means that there are *n* nodes in the cluster, *w*″*_j,i_* means the data weight of the node *i* within the *j* − *th* cluster; the general weight vector of the *j* − *th* cluster is *W_j_* = (*w*_*j*,0_, . . ., *w_j,i_*, . . ., *w*_*j,n*−1_),*i* ∈ [0, *n* − 1], *w_j,i_* means the general weight of the node *i* with the *j* − *th* cluster.

Next, we will discuss how to get the value of the three weight vector we defined before, which is the main point of this paper.

## 2. The Architecture of the Algorithms for Each Weight Vector

### 2.1. The Space Weight Vector

The data weight vector is easier to be computed than the space weight vector for its direct physical meaning of real world and easy for computer to realize. The space weight can be identified easily by people, but for computer to understand, the system has to study special algorithms. The first step we should do is to make the system to find the central node of the cluster, and then to figure out the space weight of each node to the central node according to the shortest path principle.

Generally speaking, the greater the space weight is, the nearer the node to the cluster core is, and vice versa. The node with maximum weight is the central node of the cluster namely the space cluster core. For example, Table 1 shows the space weight vector of one cluster. The weights marked * belong to the cluster member. The node with maximum weight is (2, 2), for which *W*′_(2,2)_ = 10 and so the node is the cluster space core.

**Figure 2.**
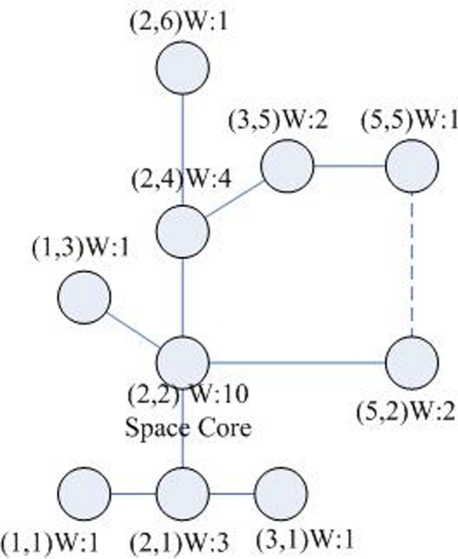
The multicast tree according to the space weight.

If we establish the multicast tree for one cluster, only consider the space weight, the tree should be as the one shown in Figure 2.

### 2.2. The Data Weight Vector

Compared with the space weight vector, the data weight vector is easier obtain, as we can just define the date weight according to the date amount on the node directly [17]. In Table 2, the data weight vector is listed, in the cluster, the weights marked * belong to the cluster member. (In this table, 1 means 1T byte data or more.)

**Figure 3.**
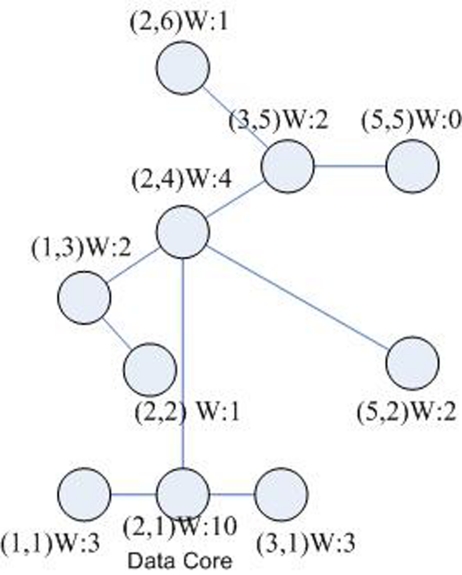
The multicast tree according to the data weight.

If we establish the multicast tree in one cluster, only consider the data weight, the tree should be in the way shown in Figure 3. But things are always not so easy, for each factor would maximize its own interest and on one hand they are in two completely separate systems, having themselves characteristics. And each factor would maximize its own interests [18]. On other hand they co-exist in the real world, and are closely relative, constraint, and drawing a balance between each other. So we should try to find the general weight vector *W*, which can synthetically consider the space factor and data factor, therefore the system can get the optimal effect based on the game and balance for both of the space and data factors.

### 2.3. The General Weight Vector

We define the general weight vector for one node as the function of space weight vector *W*′ and date weight vector *W*″, in the form of *W* = *f*(*W*′*, W*″). Now we know *W*′, *W*″, but do not know the expressions of *f*(). In other words, the function is still a black box, so we must find a way to change the black box to a white box. The relationship between the two vectors *W*′ and *W*″ can have various forms. We can start the step by discussing the most simple way: the linear relationship, which can represent the typical basic prototype of our real world. The space factors and data factors are independent with each other in the realistic sense, therefore these two vectors are linear unrelated, thus *W* = *f*(*W*′, *W*″) should be *W* = *αW*′ + *βW*″, where *α*, *β* are linear parameters.

Next, the question is how to calculate the linear parameters. We found one equation array to describe the problem for *i*-*th* cluster, based on game and balance theory illustrated next:

$$\{\begin{array}{l} \alpha_{i}+\beta_{i}=1\\ \frac{W_{i},\;W_{i}^{\prime }}{\left\|W_{i}^{\prime }\right\|}=\frac{W_{i}\cdot W_{i}^{\prime \prime }}{\left\|W_{i}^{\prime \prime }\right\|} \end{array}$$

Then the system can resolve the value of *α*, *β* by these equations, furthermore to get the general weight *W*.

Base on the weight vector *W*, we can construct the least weighted path tree next, namely the multicast tree, to transmit data, which would be the optimal path considering both of the space and data factor.

## 3. Algorithms for Game and Balance Multicast Architecture

### 3.1. Cluster Formation Algorithm

In the algorithms presented by this paper, the group members are initially split into several clusters by some management nodes (called as Rendezvous Points—RP). The cluster size is normally set as:

(1) S=(k, 3k−1)

The expression (*k*, 3*k* − 1) represents a random constant between *k* and 3*k* − 1. Like NICE, which is a hierarchical multicast technique that uses a fixed value k. The k is a constant, and in the simulation conducted in this paper, it is acceptable to use *k* = 3. The definition of cluster size is the same as the one of NICE, which is to avoid the frequent cluster splitting and merging [5]. We define the state of node that has not been assigned into any cluster as unassigned. We describe the cluster formation as follows.

The RP initially selects the left lowest end host (say U) among all unassigned members. The left lowest node is the node that has the minimum coordinates along *m* dimensions among all nodes occupied by the unassigned group members. The cluster member selection is in the dimension order around U by using the following algorithm.

**Figure 4.**
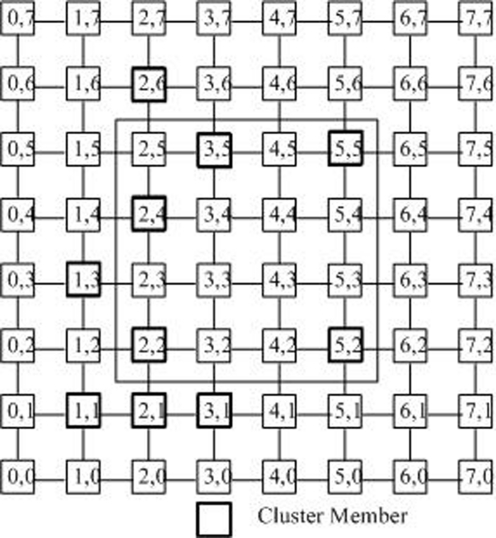
Selecting the spatial center nodes in the members of one cluster of a 2-*D* Sensor grid.

Figure 4 illustrates the spatial center nodes in a 2-*D* sensor grid. In this sensor grid, the initial left lower end host is (0, 0). According to Step 5, the RP firstly selects the end host in (0, 1) into the cluster. Because *j* = 0, Steps 8–11 are neglected. Then, the RP selects the end host in (1, 0)into the cluster by Steps 5–7. Based on Steps 8–11, the next selected cluster member is the one in (1, 1). The cluster formation guarantees that each cluster contains the closest group members in terms of static delay distance [19]. According to the results in [20], the scheme that assigns closed members into the same cluster will improve the scalability and efficiency of the multicast data transfers of the sensor grid.

**Algorithm 1:**
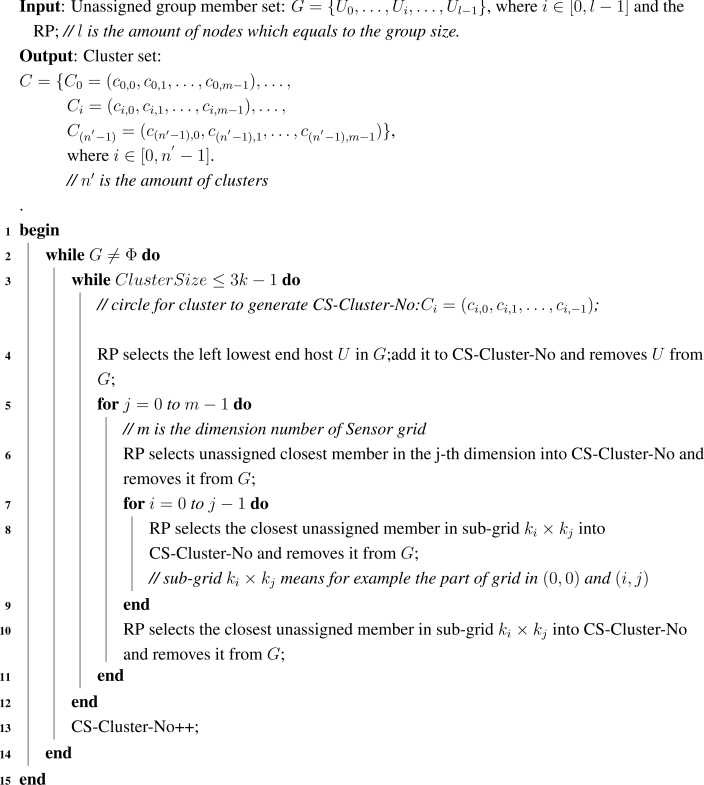
Cluster Formation

### 3.2. Relative Weight Vectors Generation Algorithm

This sub-algorithm generates two weight vectors: the space weight vector and the data weight vector. In addition, the node with the maximum space weight is named the space core, and the node with the maximum date weight is named the data core. Hence it can be divided into four steps:

#### A. To find the space center nodes as the space core ***C_i,a_*** in every cluster ***C′_i_***

Each cluster will have a spatial center node as the space core. The space core can be the root of the tree in the cluster. The following theorem provides the sufficient and necessary conditions to select a spatial core in each cluster that is optimal in terms of the minimum sum of static delay distances to all the other cluster members.

**Theorem 1** *Let U be the cluster member that occupies the node* (*u*_0_, . . ., *u_j_*, . . ., *u*_*m*−1_) *in an m-D grid and n* > *j,n* < *j and n* = *j be the number of cluster members with the j-th coordinates larger than (right nodes of j-th row), less than (left nodes of j-th row), and equal to u_j_ (the nodes just on j-th row) respectively. Then U is the spatial center node if and only if the following inequalities hold simultaneously:*

(2) $$|n_{<j}-n_{>j}|\leq n_{=j},\;j=0,\;1,\;\ldots ,m-1$$

**Proof:**(⇒):Suppose *U* = (*u*_0_, . . ., *u_j_*, . . ., *u*_*m*−1_) is a spatial center node, and then to any member *U*′ in the sensor grid, there exists *f*(*U*) ≤ *f*(*U*′). To achieve (2), we firstly considers a node *U*′ = (*u*_0_, . . ., *u*_*j*+1_, . . ., *u*_*m*−1_) and its multicast static delay distance *f*(*U*′). Given any member *U_i_* = (*u*_*i*,0_, . . ., *u_i,j_*, . . ., *u*_*i,m*−1_) and *u_j_* ≤ *u_i,j_*, the distance from *U_i_* to the end host *U* is one unit longer than the distance from *U_i_* to the node *U*′. Similarly, it can be seen that to any member *U_i_* = (*u*_*i*,0_, . . ., *u_i,j_*, . . ., *u*_*i,m*−1_) and *u_i,j_* ≤ *u_j_* the distance from *U_i_* to the end host *U* is one unit shorter than the distance from *U_i_* to *U*′. There exist (*n*_>*u_j_*_ + *n*_=*u*_*j*__) members whose *j* − *th* coordinates are larger than or equal to *u_j_*, and *n*_<*u*_*j*__ cluster members whose *j* − *th* coordinates are less than *U_j_*, then it can be concluded that $0\leq f(u^{\prime })-f(u)=\sum_{j=0}^{n^{\prime }}\;(d(u^{\prime },\;u_{j})-d(u,\;u_{j}))=n_{>U_{j}}+n_{=U_{j}}-n_{<U_{j}}\to n_{<U_{j}}-n_{>U_{j}}\leq n_{=U_{j}}$. By comparing *f*(*u*_0_, . . ., *u*_*j*−1_, . . ., *u*_*m*−1_) with *f*(*U*) in the same way as above, the inequality of 2 can be achieved.

(⇐): It is easy to demonstrate that if (2) is violated, and then *U* cannot be the spatial center nodes. Assume *n*_>*u*_*j*__ − *n*_<*u*_*j*__ > *n*_=*u*_*j*__, then *n*_>*u*_*j*__ > *n*_<*u*_*j*__ + *n*_=*u*_*j*__. Similarly, this paper firstly considers a node *U*′ = (*u*_0_, . . ., *u*_*j*+1_, . . ., *u*_*m*−1_) and its multicast static delay distance *f*(*U*′). Given any member *U_i_* = (*u*_*i*,0_, . . ., *u_i,j_*, . . ., *u*_*i,m*−1_) and *u_j_* ≤ *u_i,j_* the distance from *U_i_* to the end host U is one unit longer than the distance from *U_i_* to *U*′. Similarly, it can be seen that to any member *U_i_* = (*u*_*i*,0_, . . ., *u_i,j_*, . . ., *u*_*i,m*−1_) and *u_i,j_* ≤ *u_j_* the distance from *U_i_* to the node *U* is one unit shorter than the distance from *U_i_* to *U*′. $f(u)-f(u^{\prime })=\sum_{i=0}^{n^{\prime }}\;(d(u,\;u_{i})-d(u^{\prime },\;u_{i}))=n_{<u_{j}}+n_{=u_{j}}-n_{>u_{j}}>0\to f(u)>f(u^{\prime })$. Therefore the distance from *U* to these end hosts is larger than some other end hosts, which is a desired contradiction.

The physical meaning of the theory is obvious. Firstly, we process on *X* axis. For example *N*_=2_ = 4, namely there are 4 nodes just on of second row: (2, 6), (2, 4), (2, 2), (2, 1); *N*_<2_ = 2, namely there are 2 nodes in the left of second row: (1, 3), (1, 1); *N*_>2_ = 4, namely there are 4 nodes in the right of second row (3, 5), (3, 1), (5, 5), (5, 2), so |*n*_<2_ − *n*_>2_| ≤ *n*_=2_. Thus *N*_=2_ is satisfied coordinates on *X* axis. On other hand, *N*_=3_ = 2, including (3, 5), (3, 1); *N*_<3_ = 6, including (2, 6), (2, 4), (2, 2), (2, 2), (1, 3), (1, 1); *N*_>3_ = 2, including (5, 5), (5, 1), so |*n*_<3_ − *n*_>3_| ≥ *n*_=3_. Thus *N* = 3 is not satisfied coordinates. In the same way, we can do it again on *Y* axis. Then we can find the (2, 2) is the space central node, namely the space core of the cluster.

#### B. To calculate the space weight vector of every node ***W*′*_i,j_***

**Figure 5.**
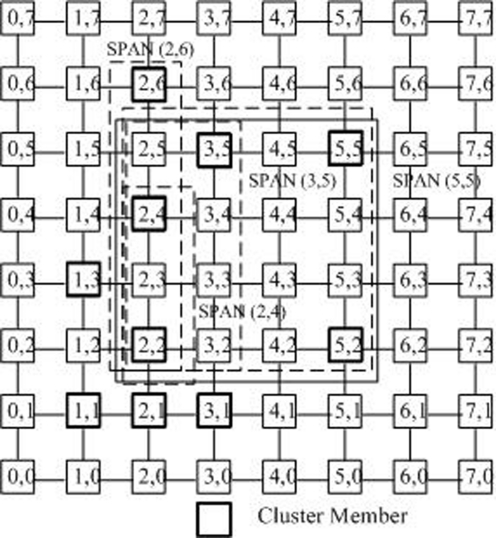
Shortest path area nodes (SPAN) in a 2-*D* Sensor grid, for example: The node (2,4) is 4 node’s Shortest Path Area Nodes (SPAN): (2,6), (3,5), (5,5), (2,4).

At the beginning of this discussion, it can be presumed that the system establishes a multicast tree to transfer data packet, which choose the space core as the root and organize the architecture according to the space weight vector [21]. It is anticipated that the tree should maximize the sharing of link utilization within the clusters so that the rest of the links may be used for other traffic [22]. Our approach is to connect all the members according to (1) the branch on the tree between two adjacent members is the shortest path in the cluster, (2) the total number of links on the tree should also be minimized. Before discussing the algorithm, it is necessary to define the following terminologies (using a 2-*D* cluster as the model):

1. **Shortest path area nodes (SPAN):** For any two nodes (*x*_0_, *y*_0_) and (*x*_1_, *y*_1_), let *X_min_* = *min*{*x*_0_, *x*_1_}, *X_max_* = *max*{*x*_0_, *x*_1_}, *Y_min_* = *min*{*y*_0_, *y*_1_} and *Y_max_* = *max*{*y*_0_, *y*_1_}. They uniquely define a rectangle area [*x*_0_, *y*_0_] × [*x*_1_, *y*_1_]. Each node (*x*, *y*) in [*x*_0_, *y*_0_] × [*x*_1_, *y*_1_], which is on one of the shortest paths between (*x*_0_, *y*_0_) and (*x*_1_, *y*_1_), so it is called the shortest path area nodes (SPAN) between (*x*_0_, *y*_0_) and (*x*_1_, *y*_1_).
2. **SPAN nodes of a cluster member:** When the tree is built in the cluster with the size of *n*, all nodes *C_j_*(*x_j_*, *y_j_*) in the SPAN area [*x*_0_, *y*_0_] × [*x_i_*, *y_i_*] from the core (i.e., the root of the tree)c*(x*, y*) to a cluster member *c_i_*(*x_i_*, *y_i_*)(*i* ∈ [0, *n* − 1])can be regarded as the SPAN nodes of *c_i_*. Take Figure 5 as an example. Assume that the core is in the node (2, 2). All nodes in [2, 2] × [5, 5]are the SPAN nodes of this cluster member.
3. **The space weight of the node:** A node may be the SPAN node of several *k* cluster members. If a node is the SPAN node of *k* cluster members, this node is assigned the weight of *k*. Table 1 gives the space weights of all nodes in Figure 4. Taking the node (2, 4) as an example, as show in Figure 5. The node (2, 4) is 4 node’s Shortest Path Area Nodes (SPAN): (2, 6), (3, 5), (5, 5), (2, 4), because it is in the Shortest Path Area of these nodes. Therefore its weight 4 means that 4 cluster members may pass through node (2, 4) to the cluster core (2, 2) by the shortest paths. Apparently, the weight of (2, 2) is 10.

In general, if the space weight of the node is *k*, it means that there are *k* nodes which must pass this node to the space core to send packets, which represent the degree near the center. The greater the space weight is, the nearer the node to the cluster core is, and vice versa.

#### C. To find the data weight ***W*″** of every node in cluster ***C_i_***

After figuring out the space weight vector *W*′*_i,j_*, the system can easily get the data quantity in every node, namely generating the data weight, because the data quantity vector is determinant, as shown in Table 2.

#### D. To find the maximal data quantity node in ***W*″*_i,j_*** as the data core ***c_i,b_*** in every cluster

According to the Table 2, because the node *B*(2, 1) is the maximal value 10, it is the data core.

The relatively Weighted Vectors Generation algorithm in the *m*-*D* Sensor grid is given in Algorithm 2.

**Algorithm 2:**
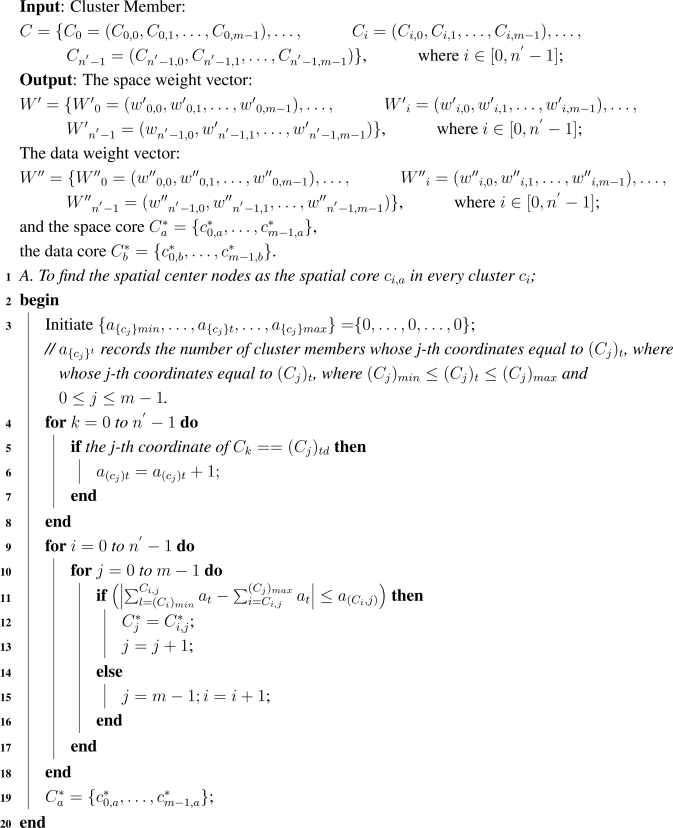

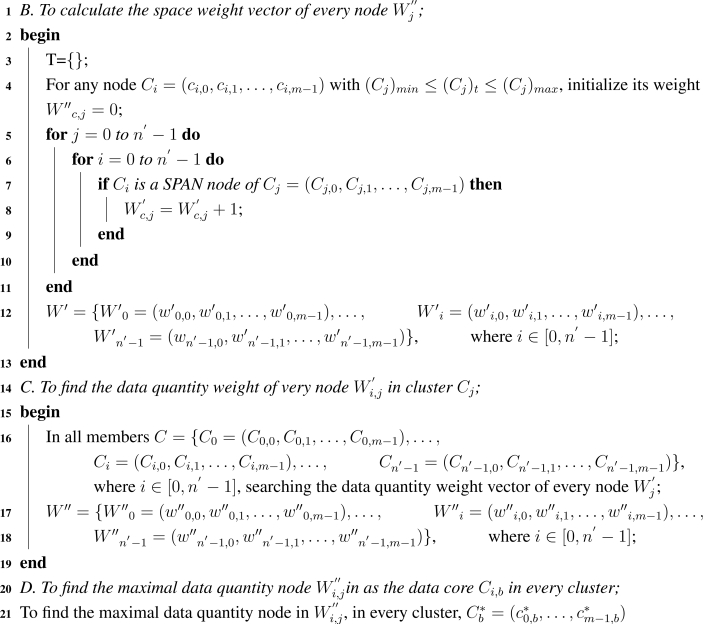
Relative Weighted Vectors Generation

In Algorithm 2, Steps 4–7 can be executed in time O(n). Steps 9–18 can be improved by using binary searching algorithm that yields an O(ln(n)) complexity. But for the brevity of discussion, it is necessary to keep the linear search algorithm here. The algorithm may find out multiple spatial central nodes, but just choose one of them at random and others can be back-ups. Figure 4 illustrates the spatial center nodes (2, 2) selection in one cluster of 2-*D* sensor grid. It is known that the spatial center node should be in the area [1, 1] × [5, 6]. Therefore, it can be checked that the spatial center node’s *x* coordinate must be 2 while *y* coordinate could be 2 or 3 for *f*((2, 2)) = *f*((2, 3)) = 26. Node (2, 2) is the member and is preferential (2, 3).

In the sensor grid, the data quantities of nodes are very different, for instance, about 20% nodes process 80% of the data quantity of the whole system. These nodes are very important in the multicast data transfer, therefore when researching the algorithm in multicast, the node’s data quantity weight *W*″ should be taken into account. On the other hand, the spatial nodes weight *W*′ in the Algorithm 3 (Least Weighted Path Tree Generation) should be noticed too. The relationship between the two vectors is a very challenging question, we tried in this paper to begin from the linear relative between them.

In general, the Relative Weighted Vectors Generation algorithm, finds the spatial core *c_i,a_* in every cluster *C_i_*, calculates the space weight of every node *W*′*_i,j_*, searches the data quantity weight of the very node *W*″*_i,j_* in cluster *C_i_*, and finally finds the data core *c_i,b_*.

### 3.3. Least Weighted Path Tree Generation Algorithm

After the Relative Weighted Vectors Generation algorithm generates the space weight vector *W*′ and the data weight vector *W*″, and the spatial core *c_i,a_* and the data core *c_i,b_*, the Least Weighted Path Tree Generation algorithm wants to combine the two old weight vectors *W*′ and *W*″ to a new weight vector *W*. Now the system just knows *W* = *f*(*w*′, *w*″), but does not know the expression of the *f*(). As we mentioned in Section 2.3., we used the linear form: *W* = *αW*′ + *βW*″. After that the sub-algorithm builds binary simple equations, resolves linear parameters *α*, *β*, generates new weight vector *W*. At last generates the least weighted path tree as hierarchical multicast tree. The sub-algorithm can be divided into 5 steps:

#### A. To define the weights of the nodes

(3) $$W_{i,j}=\alpha_{i}\;W_{i,j}^{\prime }+\beta_{i}\;W_{i,j}^{\prime \prime }$$

*W_i,j_* : The weights of the nodes;
*α_i_*, *β_i_*: Linear relation modulus, *α_i_*, *β_i_* ∈ *r*; *α_i_*, *β_i_* ≥ 0, as *α_i_*, *β_i_* < 0 nonsense;
*W*′*_i_* : The space weight vector.
*W*″*_i_*: The data weight vector.

#### B. The linear relation modulus of the weight of the node satisfied

(4) $$\alpha_{i}+\beta_{i}=1;0<\alpha_{i},\;\beta_{i}<1;\alpha_{i}\in r.$$

**Theorem 2** *If, two linear no-relationship vector W′_i_, W″_i_, their linear combination W_i,j_* = *α_i_W′_i,j_* + *β_i_W″_i,j_, α_i_, β_i_ is linear relation modulus, α_i_, β_i_* ∈ *r, α_i_, β_i_* ≥ 0*, then following express is satisfied:*

$$\alpha_{i}+\beta_{i}=1,\;0<\alpha_{i},\;\beta_{i}<1,\;\alpha_{i},\;\beta_{i}\in r$$

**Proof:** *The rationality of the equation is obvious:*

*Let* $W_{i,j}^{*}=\alpha_{i}^{*}\;W_{i,j}^{\prime }+\beta_{i}^{*}\;W_{i}^{\prime \prime },\;\alpha_{i}^{*},\;\beta_{i}^{*}\in r,\;\alpha_{i}^{*},\;\beta_{i}^{*}\geq 0$.
$\frac{W_{i,j}^{*}}{\alpha_{i}^{*}+\beta_{i}^{*}}=\frac{\alpha_{i}^{*}}{\alpha_{i}^{*}+\beta_{i}^{*}}\;W_{i,j}^{\prime }+\frac{\beta_{i}^{*}}{\alpha_{i}^{*}+\beta_{i}^{*}}\;W_{i,j}^{\prime \prime }$
*Take* $W_{i,j}=\frac{W_{i,j}^{*}}{\alpha_{i}^{*}+\beta_{i}^{*}}$, $\alpha_{i}=\frac{\alpha_{i}^{*}}{\alpha_{i}^{*}+\beta_{i}^{*}}$, $\beta_{i}=\frac{\beta_{i}^{*}}{\alpha_{i}^{*}+\beta_{i}^{*}}$.
*Then W_i,j_* = *α_i_W′_i,j_* + *β_i_W*″*_i,j_, and α_i_* + *β_i_* = 1, 0 < *α_i_*, *β_i_* < 1, *α_i_*, *β_i_* ∈ *r*

#### C. The space factor and data factor are game and balance with each other, the game balance point is

(5) $$\frac{W_{i}\cdot W_{i}^{\prime }}{\left\|W_{i}^{\prime }\right\|}=\frac{W_{i}\cdot W_{i}^{\prime \prime }}{\left\|W_{i}^{\prime \prime }\right\|}$$

**Theorem 3** *If two linear no-relationship vector, W*′*_i_* = (*w*′_*i*,0_, . . ., *w*′*_i,j_*, . . ., *w*′_*i,m*−1_), *W*″*_i_* = (*w*″_*i*,0_, . . ., *w*″*_i,j_*, . . ., *w*″_*i,m*−1_) *their linear combination W_i_* = (*w*_*i*,0_, . . ., *w_i,j_*, . . ., *w*_*i,m*−1_), *and W_i_* = *α_i_W′_i_* + *β_i_W″_i_*, *α_i_, β_i_ is linear relation modulus, α_i_, β_i_* ∈ *r, α_i_, β_i_* ≥ 0. *The game balance point of W*′*_i_ and W*″*_i_ is*

$$\frac{W_{i}\cdot W_{i}^{\prime }}{\left\|W_{i}^{\prime }\right\|}=\frac{W_{i}\cdot W_{i}^{\prime \prime }}{\left\|W_{i}^{\prime \prime }\right\|}$$.

**Figure 6.**
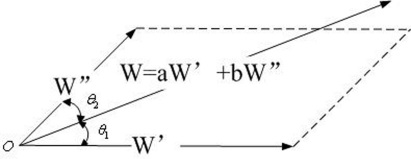
The relationship of *W_i_*, *W*′*_i_* and *W*″*_i_*.

**Proof:** *Because W*′*_i_* = (*w*′_*i*,0_, . . ., *w*′*_i,j_*, . . ., *w*′_*i,m*−1_), *W*″*_i_* = (*w*″_*i*,0_, . . ., *w*″*_i,j_*, . . ., *w*″_*i,m*−1_) *their linear combination W_i_* = (*w*_*i*,0_, . . ., *w_i,j_*, . . ., *w*_*i,m*−1_), *and W_i_* = *α_i_W′_i_* + *β_i_W″_i_, as show in Figure 6.*

*Because* $\cos\theta_{1}=\frac{W_{i}\cdot W_{i}^{\prime }}{\left\|W_{i}\right\|\cdot \left\|W_{i}^{\prime }\right\|}$, $\cos\theta_{2}=\frac{W_{i}\cdot W_{i}^{\prime }}{\left\|W_{i}\right\|\cdot \left\|W_{i}^{\prime }\right\|}$, *we want to find the game and balance point to make θ*_1_=*θ*_2_, *then cosθ*_1_=*cosθ*_2_.

*On other words, the game balance point between W′_i_ and W″_i_ should be the point that has the equal correlation coefficient to each of the vectors. That is to say:* $\frac{W_{i}\cdot W_{i}^{\prime }}{\left\|W_{i}\right\|\cdot \left\|W_{i}^{\prime }\right\|}=\frac{W_{i}\cdot W_{i}^{\prime \prime }}{\left\|W_{i}\right\|\cdot \left\|W_{i}^{\prime \prime }\right\|}$, *then we get:* $\frac{W_{i}\cdot W_{i}^{\prime }}{\left\|W_{i}^{\prime }\right\|}=\frac{W_{i}\cdot W_{i}^{\prime \prime }}{\left\|W_{i}^{\prime \prime }\right\|}$.

Combining (4) and (5), the paper builds the liner binary simple equations:

$$\{\begin{array}{l} \alpha_{i}+\beta_{i}=1\\ \frac{W_{i}\cdot W_{i}^{\prime }}{\left\|W_{i}^{\prime }\right\|}=\frac{W_{i}\cdot W_{i}^{\prime \prime }}{\left\|W_{i}^{\prime \prime }\right\|} \end{array}$$

For *W_i_* = *α_i_W′_i_* + *β_i_W″_i_*

$$\begin{array}{l} \frac{(\alpha_{i}W_{i}^{\prime }+\beta_{i}W_{i}^{\prime \prime })\cdot W_{i}^{\prime }}{\left\|W_{i}^{\prime }\right\|}=\frac{(\alpha_{i}W_{i}^{\prime }+\beta_{i}W_{i}^{\prime \prime })\cdot W_{i}^{\prime \prime }}{\left\|W_{i}^{\prime \prime }\right\|}\;\text{then}\\ (\alpha_{i}W_{i}^{\prime }+\beta_{i}W_{i}^{\prime \prime })\;\cdot \;W_{i}^{\prime }\;\cdot \;\left\|W_{i}^{\prime \prime }\right\|=(\alpha_{i}W_{i}^{\prime }+\beta_{i}W_{i}^{\prime \prime })\;\cdot \;W_{i}^{\prime \prime }\;\cdot \;\left\|W_{i}^{\prime }\right\| \end{array}$$

For *α_i_* + *β_i_* = 1, then

$$\begin{array}{l} \alpha_{i}=\frac{\left\|W_{i}^{\prime \prime }\right\|}{\left\|W_{i}^{\prime }\right\|+\left\|W_{i}^{\prime \prime }\right\|}=\frac{\sqrt{\sum_{j=0}^{m-1}\;{w_{i,j}^{\prime \prime }}^{2}}}{\sqrt{\sum_{j=0}^{m-1}\;{w_{i,j}^{\prime }}^{2}}+\sqrt{\sum_{j=0}^{m-1}\;{w_{i,j}^{\prime \prime }}^{2}}}\\ \beta_{i}=\frac{\left\|W_{i}^{\prime }\right\|}{\left\|W_{i}^{\prime }\right\|+\left\|W_{i}^{\prime \prime }\right\|}=\frac{\sqrt{\sum_{j=0}^{m-1}\;{w_{i,j}^{\prime }}^{2}}}{\sqrt{\sum_{j=0}^{m-1}\;{w_{i,j}^{\prime }}^{2}}+\sqrt{\sum_{j=0}^{m-1}\;{w_{i,j}^{\prime \prime }}^{2}}} \end{array}$$

So: 0 < *α_i_*, *β_i_* < 1, *α_i_*, *β_i_* ∈ *r*

According to the above data table, the algorithm figures out *α_i_* = 0.53, *β_i_* = 0.47.

#### D. To get the weight vector and choose the maximum value node as the cluster core

According to the above all, the algorithm gets the weight vector (as Table 3) and chooses the maximum value node as the cluster core $C^{*}=(c_{0}^{*},\ldots ,c_{i}^{*},\ldots ,c_{m-1}^{*})$, in this cluster it chooses (2, 1) as cluster core *c_i_*.

#### E. Path Weight:

Given a shortest path, the path weight is the sum of all on-path node weights. For example, the weight of path < (2, 2), (2, 3), . . ., (2, 5), . . ., (5, 5) > is ∑*_i_ α_i_W*′*_i_* + ∑*_i_ β_i_W*″*_i_*.

Let the cluster with *n*′ members be:

$$\begin{array}{lll} C & = & \{C_{0}=(c_{0,0},\;c_{0,1},\ldots ,c_{0,m-1}),\ldots ,\\ & & C_{i}=(c_{i,0},\;c_{i,1},\ldots ,c_{i,m-1}),\ldots ,\\ & & C_{n^{\prime }-1}=(c_{n^{\prime }-1,0},\;c_{n^{\prime }-1,1},\ldots ,c_{n^{\prime }-1,m-1})\} \end{array}$$

After obtaining the space weight vector *W*′, the data weight vector *W*″, the spatial core *c_i,a_* and the data core *c_i,b_*, the paper will calculate the weight vector *W* and the cluster core $c_{j}^{*}$.

The main idea of the least weighted path tree generation algorithm can be sketched as follows. After obtaining the space weight vector and the data quantity weight vector, we tried to find a new vector composed by linear combination of the two old ones. And it builds binary simple equations between them, seeks linear parameters. Then they generate a least weighted path tree, namely multicast tree. The least weighted path tree generation algorithm is shown in Algorithm 3.

**Algorithm 3:**
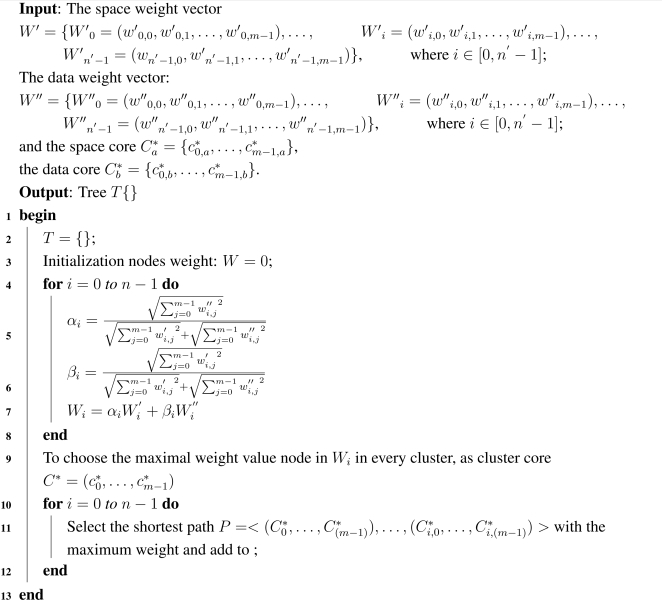
Least Weighted Path Tree Generation.

For example: in the cluster, the spatial center node is *A*(2, 2), and the maximal data quantity node is *B*(2, 1). For the requirement of practice, in this cluster, let $\frac{W_{i}\cdot W_{i}^{\prime }}{\left\|W_{i}^{\prime }\right\|}=\frac{W_{i}\cdot W_{i}^{\prime \prime }}{\left\|W_{i}^{\prime \prime }\right\|}$, and *α_i_* + *β_i_* = 1. To resolve the liner binary simple equations, the paper gets the liner relation modulus *α_i_*, *β_i_* = *f*(*W*′*_i_, W*″*_i_*), then gets *α_i_* = 0.53, *β_i_* = 0.47. At last, the algorithms attain new weight vector *W*.

Notice that since the dimension of the space weight vector and the data quantity weight vector may be different, the data quantity weight vector may stem from a function of the nature data quantity.

### 3.4. Multicast Routing Algorithm

Firstly, the network is partitioned into clusters by some regular Sensor grid area; after group members are divided into different clusters, a tree is built to connect the cluster members in each cluster; at last, the connection among different clusters is done through hooking the tree roots.

**Algorithm 4:** Multicast routing for group G

**1** Hence *s* sends its multicast messages to its cluster core *c*_0_. **2** The cluster core *c*_0_ sends them to all other cores *c_i_* **3** The cluster core *c*_0_ routes the multicast packets to its own cluster members along the cluster tree. **4** At the same time, all cluster cores *c_i_*, upon receiving the multicast messages, transmit them along the cluster trees to all cluster members *m_i_* within the clusters.

## 4. Performance Evaluation

### 4.1. The Model of Simulation

We evaluated the newly-proposed optimal hierarchical multicast algorithms with the simulation developed by C++ [23] and run by a group of 40 IBM double cores PC. And we chose three multicast routing approaches for 2-*D* Sensor grid used for the performance testing and comparison: SPACE which just considers space factor; DATA which just considers data factor; and GBMASG which synthetically considers space and data factors. Moreover in multicast technology, for the space factor, there are several multicast routing algorithms, for example Double-Channel XY Multicast Wormhole Routing (DCXY) [20], Dual-Path Multicast Routing (DPM) [24], RCWP, OCXYP, RCXYP, etc. Among these approaches, the DCXYP is the most popular, the routing way just as our SPACE approach. Here we use DATA aprcoch according to data weight vector to generate least weight path tree.

In the simulation environment, the network topology used in the simulation is a 2-*D* Sensor grid. The bandwidth of each link is 10 Mbps [25]. During the simulation, 1,000 and 1,000,000 multicast packets are randomly generated as time seed and the average size of the packets is 2,400 bytes so that the average time to transmit a packet on the defined link is about 1 ms [26]. The following two metrics are employed to evaluate these multicast schemes: **Average multicast delay:** Defining the message multicast delay at a node as the sum of the routing delay, queuing delay and transmission delay. The average multicast delay AD is computed by

(6) $$\mathrm{AD}=(\sum_{i=0}^{n-1}\;d(s,\;u_{i}))/n$$

where *d*(*s*, *u_i_*) is the packet delay from the source *s* to the member *u_i_* and *n* is the group size.

**Number of links used:** It refers to the total number of links used in G in order to multicast the messages to all group members.

### 4.2. The Result of Simulation

**Figure 7.**
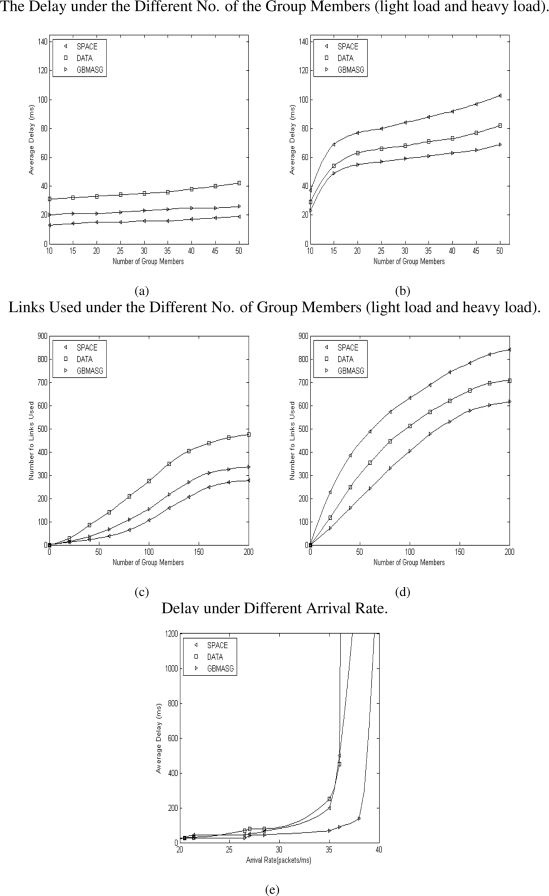
Simulation results for SPACE, DATA and our GBMASG.

The average delay metric under the light and heavy load of network is shown in Figure 7(a),(b). The link usage for different algorithms under the light and heavy load of network is shown in Figure 7(c),(d). It can be seen that the average delay increases with the increase of the network load Figure 7(e) and once it surpasses one point the the average delay will increase rapidly. From these simulation results, it can be obtained the following observations:

1. Under the light load circumstance, the delay is mainly decided by the distance from the source to the group members Figure 7(a). The SPACE approach always transmits multicast packets to group members along the shortest paths from the source to the group members, therefore it achieves the best delay performance among the other systems when the network is lightly loaded. When the traffic is low, GBMASG achieves as good delay performance as SPACE, but with a little bit difference. DATA’s performance is not very good for light load.
2. Under a heavy load circumstance, the delay is mainly decided by the source of the data quantity, and certainly relates to the space of the nodes too Figure 7(b). In the sensor grid circumstance, a majority of data quantity will concentrate in minor nodes. Now that SPACE just generate the multicast tree according to space factor, so that the delay increases a little rapidly in the mass data quantity. Our approach GBMASG synthetically considers both data and space factors, so that it gets the best result. DATA achieves the quite well delay performance almost as GBMASG here Figure 7(b).
3. Figure 7(c),(d) show the average number of links used by these approaches. In general the number of links will be increased with the number of the group members. At light load, the delay is mainly decided by the distance from the source to the group members Figure 7(c). Therefore SPACE achieves the least the number of links compared with other approach when the network is lightly loaded. GBMASG is in middle and approaching SPACE, but DATA is not very good.
4. At heavy load, the number of links is mainly decided by both the source of the data quantity and the space of the node Figure 7(d). So that for SPACE the number of links increases rapidly in the mass data quantity, and the DATA is much better, at last our approach GBMASG get the least the number of links.
5. Figure 7(e) shows that the delay increases as the packet arrival-rate increases. The system saturation points for SPACE, DATA and GBMASG are about 35, 36 and 38 packets/ms respectively. Our algorithm GBMASG achieves the maximum throughput.

It reveals that under the same condition, GBMASG obtains the best balance over the performance parameters, i.e., the less resource a system consumes, the higher the throughput and the shorter the delay under heavy traffic load [27]. The GBMASG is especially suitable for sensor grid with large data quantity.

## 5. Conclusions and Future Work

### 5.1. Conclusions

A sensor grid integrates wireless sensor networks with grid infrastructures to enable real-time sensor data collection and the sharing of computational and storage resources for sensor data processing and management. It is an enabling technology for building large-scale infrastructures. When the system constructs the hierarchical tree, it should consider not only the factor of the space, but also the data quantity. Their relationship is game and balance. We tried to draw an elaborate balance between them, and uses the basic idea to construct the hierarchical multicast tree in this paper.

The network is partitioned into clusters in terms of the static delay distance of Sensor grid area. After group members are initially scattered into different clusters, a tree is built to connect the cluster members with each other. The connection among different clusters is done through hooking the tree roots. To construct such architecture, a set of novel algorithms based on the *m*-*D* sensor grid are composed of four sub-algorithms:

1. Cluster formation algorithm. It divides the group members into different clusters in terms of static delay distance;
2. Relative weight vectors generation algorithm. It figures out two weight vectors: the space weight vector *W*′ and the data weight vector *W*″. In addition, it generates the spatial core *C_i,a_*, which is the node with the maximum space weight node, and the data core *C_i,b_*, which is the node with the maximum space weight node also.
3. Least weighted path tree algorithm. After the Relative Weighted Vectors Generation algorithm generates the space weight vector *W*′ and the data weight vector *W*″, and the data core *C_i,a_* and the spatial core *C_i,b_*, the sub-algorithm wants to combine the two old weight vectors *W*′ and *W*″ to a new weight vector *W*. But the system just knows *W* = *f*(*W*′, *W*″), but does not know the expression of the *f*(). The relationship between the two vectors *W*′ and *W*″ can have various forms. We can start the step by discussing the most simple way: the linear relationship, which can represent the typical basic prototype of our real world: *W* = *αW*′ + *βW*″. After that, the sub-algorithm builds binary simple equations, resolves linear parameters *α*, *β*, generates new weight vector *W*. At last generates the least weighted path tree as multicast tree.
4. Multicast Routing Algorithm. Firstly, the network is partitioned into clusters in terms of some regular sensor grid area. After group members are initially scattered into different clusters, a tree is built to connect the cluster members within each cluster. At last, the connection among different clusters is done through hooking the tree roots to implement the inter-cluster routing.

### 5.2. Future Work

#### 1. To extend the multicast algorithm from **2-*D*** to **3-*D*** space

At present, the algorithm in this paper mostly focuses on optimal data transfer strategy in 2-*D* space. The reason is that recently mostly applications are still limited within 2-*D*. With the development of technology, Sensor grid architecture steps into outer space, into 3-*D*.

#### 2. To extend the data quantity weight vector ***W*″**

In the paper the space weight vector is complex, but the data quantity weight vector is definite, therefore the system can gain it directly. However, in some situation the data quantity could be changing, so that we should study special sub-algorithm for it. For example, in Pervasive Computing, the data quantity weight vector is not definite, therefore it is a function: *W*″ = *f*(*x*).

#### 3. To discuss the non-linear relationship of two vectors ***W*′** and ***W*″**

Moreover, we just discussed about the liner relationship of two vectors in this paper. On the other level, the above method can just resolve the linear relationship of two vectors. But, in practice, the system sometimes does not transform linearly but exponentially. The paper can resolve one exponential equation in the situation. For example, we can define the nodes weights as $W_{i,j}=\sqrt{{\left(\alpha_{i}W_{i,j}^{\prime }\right)}^{2}+{\left(\beta_{i}W_{i,j}^{\prime \prime }\right)}^{2}}$. In the meantime the system can use some other methods to control, such as controlling theory method, differential method, etc.

#### 4. To extend to 3 vectors correlation

Furthermore, after discussed two vectors correlation: the space weight vector and data weight vector, the paper can easily be extended to three weight vectors correlation, for instance the economy weight vector. In this situation, the costs of server in different place vary, therefore the clients prefer the node with cheaper cost. Therefore the system should take the economy factor into account, while constructing multicast tree. The three weight vectors are game and balance with each other. The relationship of the weight should be *W_i,j_* = *α_i_W*′*_i,j_* + *β_i_W*″*_i,j_* + *λ_i_W*″′*_i,j_*. And it can easily extend the equation array as

(7) $$\{\begin{array}{l} \alpha_{i}+\beta_{i}+\gamma_{i}=1\\ \frac{W_{i}\cdot W_{i}^{\prime }}{\left\|W_{i}^{\prime }\right\|}=\frac{W_{i}\cdot W_{i}^{\prime \prime }}{\left\|W_{i}^{\prime \prime }\right\|}=\frac{W_{i}\cdot W_{i}^{\prime \prime \prime }}{\left\|W_{i}^{\prime \prime \prime }\right\|} \end{array}$$

#### 5. To extend to N-vectors correlation

After discussing two and three vectors correlation, the algorithm can be extended to N-vectors correlation. Because in the realistic world, people should consider a number of factors while constructing the multicast tree, for example: space, data, economy, politics, military, etc. In this mode, every factor is a vector. As long as the physic mean of different factor is independent, the weight vector is linear (non-relative). Even if the two vectors are linear related, there are the ways to turn it to be linear (non-relative). All these factors can be denoted by a series of weight vectors. All of them game and balance with each other.

The relationship of the weight can be defined as $W_{i,j}=\alpha_{i}^{\left(1\right)}W_{i,j}^{\left(1\right)}+\ldots +\alpha_{i}^{\left(k\right)}W_{i,j}^{\left(k\right)}+\ldots +\alpha_{i}^{\left(n\right)}W_{i,j}^{\left(n\right)}$. And the equation can be extended to

$$\{\begin{array}{l} \alpha_{i}^{\left(1\right)}+\ldots +\alpha_{i}^{\left(k\right)}+\ldots +\alpha_{i}^{\left(n\right)}=1\\ \frac{W_{i}\cdot W_{i}^{\left(1\right)}}{\left\|W_{i}^{\left(1\right)}\right\|}\ldots =\frac{W_{i}\cdot W_{i}^{\left(k\right)}}{\left\|W_{i}^{\left(k\right)}\right\|}\ldots =\frac{W_{i}\cdot W_{i}^{\left(n\right)}}{\left\|W_{i}^{\left(n\right)}\right\|} \end{array}$$

It can be solved by mathematical induction.

Moreover, these factors are based on linear non-relationship condition, so their cardinal number is accountable infinite. But in realty many factors are linear relationship, so that we can turn these to be linear non-relationship. If factors are of linear relationship, then their cardinal number is unaccountable infinite.

## Figures and Tables

**Table 1.** The space weight vector *W*′ in one cluster, the weights marked * belong to the cluster member.

Y=6	0	1*	0	0	0
Y=5	0	3	2*	1	1*
Y=4	0	4*	2	1	1
Y=3	1*	5	2	1	1
Y=2	2	10*	4	2	2*
Y=1	1*	3*	1*	0	0
	X=1	X=2	X=3	X=4	X=5

**Table 2.** The data weight vector *W*″, in the cluster, the weights marked * belong to the cluster member.

Y=6	0	1*	0	0	0
Y=5	0	3	2*	1	0*
Y=4	0	5*	2	2	1
Y=3	2*	4	3	2	1
Y=2	2	1*	4	3	2*
Y=1	3*	10*	3*	0	0
	X=1	X=2	X=3	X=4	X=5

**Table 3.** The weight vector *W*″, in the cluster, the weights marked * belong to the cluster member.

Y=6	0	1.00*	0	0	0
Y=5	0	3	2.00*	1	0.53*
Y=4	0	4.47*	2	2	1
Y=3	1.47*	4	3	2	1
Y=2	2	5.79*	4	3	2.00*
Y=1 hline	1.94* X=1	6.27* X=2	1.94* X=3	0 X=4	0 X=5
